# ^18^F-FAPI-04 PET/CT in the evaluation of ground-glass nodules less than 3 cm in diameter comparing to ^18^F-FDG PET/CT

**DOI:** 10.3389/fonc.2025.1605678

**Published:** 2025-11-10

**Authors:** Zhehao Lyu, Jianing Liu, Wei Han, Shibo Guo, Chunyu Duan, Lili Liu, Hongyue Zhao, Yexin Su, Haibo Wang, Qi Zhang, Lin Tian, Peng Fu

**Affiliations:** 1 The Department of Nuclear Medicine, The First Affiliated Hospital of Harbin Medical University, Harbin, Heilongjiang, China; 2 The Department of Nuclear Medicine, The Second Affiliated Hospital of Harbin Medical University, Harbin, Heilongjiang, China; 3 The Department of Computed Tomography (CT), The Second Affiliated Hospital of Harbin Medical University, Harbin, Heilongjiang, China; 4 The Department of Radiology, The First Affiliated Hospital of Harbin Medical University, Harbin, Heilongjiang, China; 5 The Department of Pathology, The First Affiliated Hospital of Harbin Medical University, Harbin, Heilongjiang, China

**Keywords:** early stage adenocarcinoma, ground-glass nodules, FAPI PET/CT, FDG PET/CT, cancer-associated fibroblast

## Abstract

**Background:**

This study aimed to evaluate the clinical usefulness of fluorine ^18^F−FAPI-04 PET/CT in diagnosing ground-glass nodules that are less than 3 cm in diameter in comparison with fluorine ^18^F-FDG PET/CT.

**Methods:**

Prospective analysis of ^18^F-FAPI-04 PET/CT and ^18^F-FDG PET/CT scans for 74 patients with 96 GGNs less than 3 cm from 09/2021 to 10/2024 were analyzed. ^18^F-FAPI-04 imaging was performed in patients with ^18^F-FDG PET/CT within a week. The images, parameters, and histopathological invasiveness from participants were analyzed. Tumor uptake was quantified by the maximum standard uptake value (SUV_max_), standard uptake value mean (SUV_mean_), target-to-background ratio (TBR), and the ratio of SUV_max_ of the lesion to SUV_max_ of contralateral normal lung parenchyma (SUV_index_).

**Results:**

A total of 74 patients with 96 GGNs were evaluated. Different pathological subtypes of GGNs exhibited distinct uptake values on ^18^F-FAPI-04 and ^18^F-FDG PET/CT. The invasive pathological subtypes showed higher uptakes than noninvasive subtypes, of which ^18^F-FAPI-04 PET/CT showed significantly higher uptake than ^18^F-FDG PET/CT in all subtypes of GGNs (all *P<*0.05). Notably, the optimal cut-off values for specificity of SUV_max_, SUV_mean_, SUV_index_, and TBR of ^18^F-FAPI-04 PET/CT were notably higher than those of ^18^F-FDG PET/CT.

**Conclusion:**

^18^F-FAPI-04 PET/CT is useful for detecting GGNs, outperforming ^18^F-FDG PET/CT in detecting their invasiveness. It could provide valuable guidance for early-stage adenocarcinoma diagnosis, potentially impacting patient management.

**Trial registration:**

Chinese Clinical Trial Registry, ChiCTR2100051406. Registered 23 September 2021 ‘Retrospectively registered’, https://www.chictr.org.cn/showproj.html?proj=133033

## Introduction

Among global malignant tumors, lung cancer has the highest mortality and morbidity rates (1). Early detection of pulmonary cancer is critical to management, and this often requires the use of multiple imaging modalities (2). To diagnose lung cancer accurately, a wide range of ^18^F-fluorodeoxyglucose (^18^F-FDG) PET/CT is now being used, and lung cancer screening programs are constantly being developed (3–5). However, lung adenocarcinoma with ground-glass nodules (GGNs), which is considered a very heterogeneous tumor with different histopathology and disease processes (6, 7), has little information available regarding the use of ^18^F-FDG PET/CT for early cancer detection. Despite advances in ^18^F-FDG PET/CT lung cancer screening, surgical resection of GGNs with a diameter less than 3 cm is still needed in a considerable number of cases in order to differentiate early stages of lung adenocarcinoma from benign lesions (8–10). In order to manage GGNs effectively, it is crucial to detect them accurately, and new radiotracers may be required.

Fibroblast activation protein inhibitors (FAPI) are radiotracers that have shown high promise in prior studies involving various tumor types (11). It has been confirmed that lung cancer could be diagnosed using FAPI imaging with a high maximum standardized uptake value (SUV_max_) and high contrast (12). Recent studies have also shown that FAPI PET/CT may be more accurate than FDG PET/CT when it comes to staging lung cancer, particularly when it comes to detecting metastatic disease in the brain, lymph nodes, bones, and pleurae (13). Nevertheless, previous records have neglected the use of FAPI PET/CT in detecting and predicting the growth pattern of lung adenocarcinoma with GGNs. A case report has highlighted that ^68^Ga-FAPI PET/CT showed a higher uptake of tracer than ^18^F-FDG when applied to malignant GGN, which was confirmed as invasive adenocarcinoma by postoperative pathological examination (14). Current research on molecular imaging probes targeting FAP commonly uses ^68^Ga-FAPI-04 for PET imaging. Despite the unprecedented success of ^68^Ga-FAPI-04 PET/CT in detecting primary tumors, it has its drawbacks. The broad application of Ga-labeled FAPI in clinical practice is limited due to the short half-life ^68^Ga, high costs, and insufficient availability of radionuclides from the ^68^Ge/^68^Ga generator (15). Conversely, ^18^F is the most widely used radionuclide in PET imaging, as it can be mass-produced via a cyclotron and transported over long distances (16, 17). In this prospective study, we aimed to evaluate whether FAPI is superior to FDG PET/CT in lung adenocarcinoma with GGNs less than 3 cm in diameter.

## Methods

### Study participants

This study conducted prospective ^18^F-FAPI-04 PET/CT scans and ^18^F-FDG PET/CT scans for 74 patients with 96 GGNs less than 3 cm in diameter, which were considered for surgical treatment or surgical biopsy in the First-affiliated Hospital of Harbin Medical University between September 2021 and October 2024. Written informed consent was obtained from each participant prior to ^18^F-FAPI-04 PET/CT. Following the Drug Administration Law of the People’s Republic of China, indication and labeling of the FAPI-tracers were conducted under the physician’s direct responsibility, all patients underwent FAPI PET/CT. The clinical translational study of ^18^F-FAPI-04 was approved by the Ethics Committee (approval No. 2021XJSS01) and registered in the Chinese Clinical Trial Registry (ChiCTR2100051406). The ^18^F-FAPI PET/CT scan was performed at the Nuclear Medicine Imaging Diagnosis and Treatment Center at the First Affiliated Hospital of Harbin Medical University within seven days of the ^18^F-FDG PET/CT scan after informed consent was taken.

The inclusion criteria included (a) participants with newly diagnosed GGNs who had not received any treatment before; (b) participants who had pathology results; (c) participants who had FDG PET/CT and wrote informed consent for undergoing FAPI PET/CT examinations within a week. Exclusion criteria included the presence of other primary malignancies at the time of examination, severe hepatic or renal insufficiency, or refusal to undergo FAPI scanning.

Histological diagnosis was based on the new classification of lung adenocarcinoma proposed by the International Association for the Study of Lung Cancer and classified into atypical adenomatous hyperplasia (AAH), adenocarcinoma *in situ* (AIS), microinvasive adenocarcinoma (MIA), and invasive adenocarcinoma (IAC) (18). This study merged AAH, AIS and MIA into noninvasive lesions, IAC merged into invasive lesions. Therefore, cases were divided into two groups: noninvasive lesions and invasive lesions.

### Synthesis of ^18^F-FDG and ^18^F-FAPI-04

In line with standard methods, ^18^F-FDG was manufactured by the PET/CT Department of Nuclear Medicine at Harbin Medical University’s First Affiliated Hospital (19). Following standard methodology, ^18^F-FDG was routinely synthesized at the Department of Nuclear Medicine of the First Affiliated Hospital of Harbin Medical University. An earlier description of the synthesis and labeling of ^18^F-FAPI-04 can be found here (20). Injected activities were dependent on labeling yields. According to a previous dosimetry estimate, a sufficient count rate – an effective dose of 1.6 mSv/100 MBq - an upper limit of 370 MBq regarding radiation exposure, and a lower limit of 100 MBq per exam must be achieved (21). The minimum, maximum, and median dosages for FDG and FAPI were 166.87 MBq, 336.7 MBq, and 248.64 MBq, respectively. Both ^18^F-FDG and ^18^F-FAPI-04 exhibited radiochemical purity exceeding 95%. ^18^F-FAPI-04 and ^18^F-FDG tracers met all standard criteria before being administered to humans.

### PET/CT imaging

Paired ^18^F-FDG and ^18^F-FAPI-04 PET/CT scans were obtained at intervals within 7 days of each other. Before ^18^F-FDG PET/CT scanning, participants were instructed to fast for at least 6 hours, and an average blood glucose level in peripheral blood was ensured. Before ^18^F-FAPI-04 PET/CT imaging, fasting, and blood glucose measurements were not required. Patients received an intravenous injection of ^18^F-FAPI-04 and then rested for about 1 h before imaging. Both ^18^F-FDG and ^18^F-FAPI-04 PET/CT images were acquired using a digital PET/CT scanner (uMI 780, United Imaging Healthcare). In addition to the CT scan, PET images (1 minute per bed position, 6–7 PET bed positions) were acquired after a CT scan (tube voltage: 120 kV, tube current: 100mAs). For suspected pulmonary nodules, additional local high-resolution CT was performed (tube voltage: 120kV, tube current: 180mAs). Respiratory gating calibration for the matching of pulmonary nodules. In accordance with the agency’s standard clinical protocols, the scan ranged from head to thigh. After automatic random and scattering correction, the images were reconstructed using a line-of-response reconstruction algorithm.

### Image analysis

Ground-glass nodules (GGNs) were defined and classified according to the European guidelines for the surgical management of pure ground glass opacities and part-solid nodules (22). A pure GGN (also referred to as a non-solid nodule) was defined as a homogeneous area of increased attenuation without any solid component visible on thin-section CT, whereas a part-solid GGN (also called a mixed GGN) was defined as a lesion containing both ground-glass and solid components within the same nodule. Two experienced nuclear medicine specialists analyzed PET/CT data on a consensus decision (P.F. and Z.L.). The readers were blind to the results of ^18^F-FDG PET/CT when reporting ^18^F-FAPI-04 PET/CT, including location of GGN (subpleural/perifissural and parenchymal), type of nodules (pGGN and mGGN), edge (smooth and lobulated/spiculated), internal features (bronchus sign), and adjacent structures (pleural indentation). The PET quantitative indicators included maximum standardized uptake value (SUV_max_), mean value of standardized uptake (SUV_mean_), the ratio of tracer concentration in the lesion to that in the surrounding tissue (TBR), and the ratio of SUV_max_ of lesion to SUV_max_ of contralateral normal lung paranchyma (SUV_index_). The boundary of the lesion was delineated on the axial PET scan, and this outline was automatically extended to a three-dimensional region of interest (ROI) using a 60% threshold, facilitating the calculation of the SUV_max_. For TBR and SUV_index_ background ROIs were also circular, size-matched to the lesion ROIs, and with the 60% SUV_max_ threshold. Pleural (≥1cm away), hilar (≥2cm away) and mediastinum-adjacent areas were avoided in that delineation. The corresponding lesion is diagnosed as positive when the SUV_max_ value is greater than 2.0 (23). The diameter of GGN was the longest on the standard cross-sectional lung window image. For mGGN, the diameter of the solid component was the longest diameter of the solid component on standard cross-sectional lung window images. The same experienced nuclear medicine physician evaluated these CT values of lung nodules. The attenuation values of the GGN component (CT_GGN_) and normal lung parenchyma adjacent to GGN (CT_LP_) were measured with the same size circle ROI. The difference between CT_GGN_ and CT_LP_ was calculated as CT_GGN-LP_.

### Statistical analysis

All statistical data was analyzed using SPSS software version 23.0 (SPSS, Chicago, IL, USA) and GraphPad Prism (version 8.4.2; GraphPad Software, San Diego, Calif). Normally distributed values are expressed as mean ± standard deviation (SD). Non-normally distributed values are expressed as median ± interquartile range (IQR). The analysis comparing the uptake of all lesions between FAPI and FDG PET/CT was conducted using the *Mann-Whitney U test*. Univariate analysis utilized the Cox proportional hazards regression model, while multivariate analysis employed stepwise variable selection within the Cox model. Comparing the abilities of semiquantitative PET/CT parameters for differential diagnosis of different findings was performed using receiver operating characteristic (ROC) curve analysis and the area under the ROC curve (AUC). The sensitivity, specificity and optimal cut-off values were also calculated for each parameter. Optimal cut-offs were determined based on the Youden index. In order to test the correlation between continuous and non-continuous variables, the *Spearman test* was used. The results were considered statistically significant if the *P*-value was less than 0.05.

## Results

### Patient characteristics

**Figure 1:**
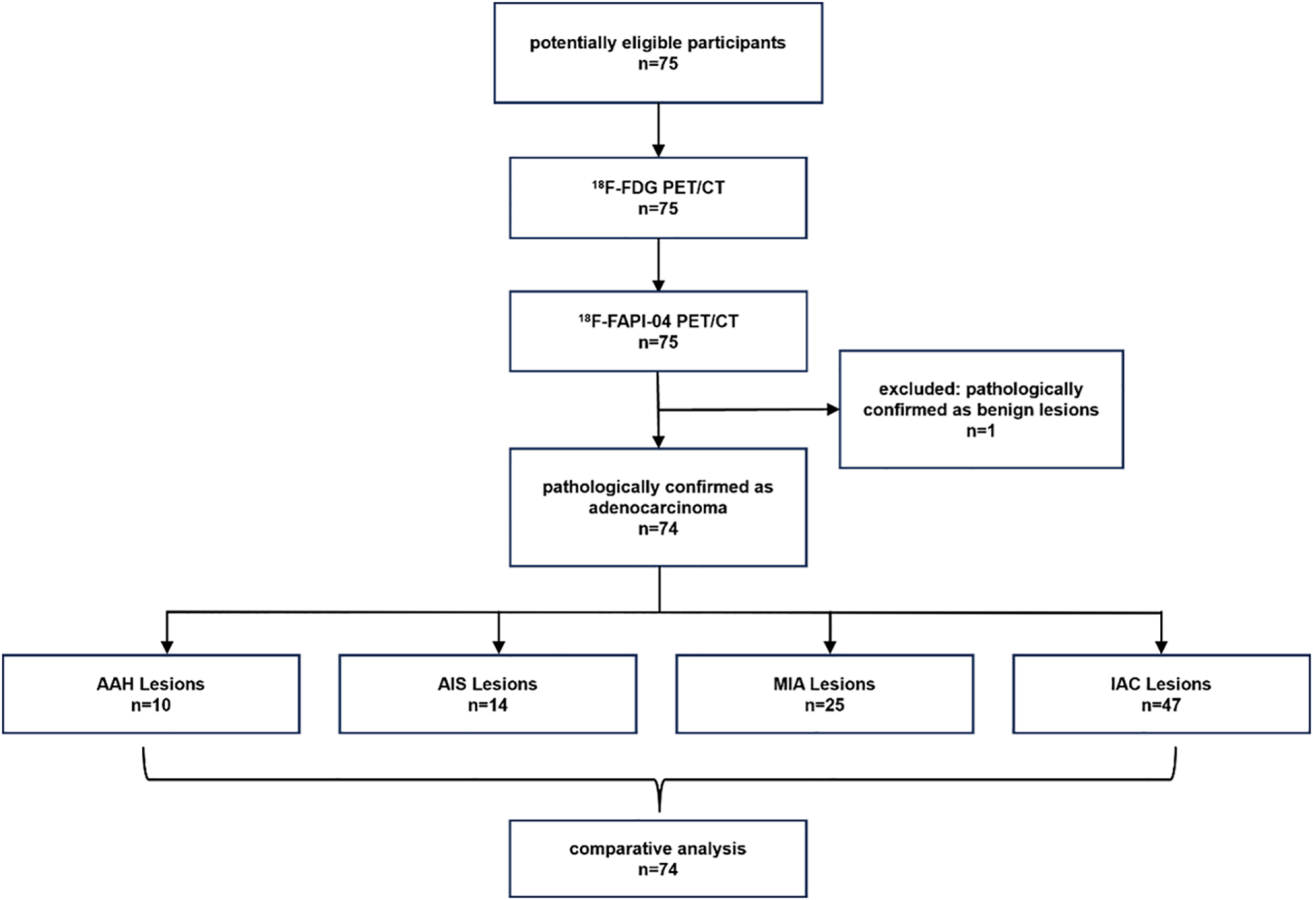
The flow diagram shows details of participants' selection and exclusions. FAPI, fibroblast-activation protein inhibitor; FDG, fluorodeoxyglucose; 18F, fluorine 18; AAH, atypical adenomatous hyperplasia; AIS, adenocarcinoma in situ; MIA, microinvasive adenocarcinoma; IAC, invasive adenocarcinoma.

From September 2021 to October 2024, 74 participants (28 men, 46 women; median age, 62 years [interquartile range: 55.5 - 69.0 years]) and 96 lesions were enrolled in the study eventually. **Figure 1** shows the study flowchart. **Table 1** summarizes these participants’ basic and clinical characteristics. Among these participants, 44 were diagnosed with pre-invasive GGNs (10 AAH, 14 AIS, and 25 MIA lesions), and 30 patients had invasive GGNs (47 IAC lesions). One case was a fibrotic lesion. All individuals were newly diagnosed with GGNs and had no previous treatment.

**Table 1:** Participants characteristics.

Characteristics	Value
No. of Participants	74
Age (y)	
Median	62
Interquartile Range	55.5 - 69
Sex	
F	46
M	28
No. of Lesions	96
AAH	10
AIS	14
MIA	25
IAC	47
Clinical features	
Family History of Lung Cancer	28
Emphysema	39
Current Smoker	10

AAH, atypical adenomatous hyperplasia; AIS, adenocarcinoma in situ; MIA, microinvasive adenocarcinoma; IAC, invasive adenocarcinoma.

### Detection rate of ^18^F-FAPI-04 PET/CT versus ^18^F-FDG PET/CT

The 75 cases, including 96 GGN lesions and one benign lesion, were diagnosed with biopsy and histopathologic examination. To further validate the superiority of ^18^F-FAPI-04 in GGN detection, we compared the quantitative uptake parameters of the two radiopharmaceuticals. In the depiction of GGNs based on SUV_max_, the detection rate was 58.3% (56 of 96) for ^18^F-FAPI-04 PET/CT and 14.6% (14.6 of 96) for ^18^F-FDG PET/CT in malignant lesions, and both tracers were negative in the benign lesion (**Table 2**). When based on SUV_index_, ^18^F-FAPI-04 demonstrated more positive lesions than that of ^18^F-FDG PET/CT (87 vs. 68). In total, ^18^F-FAPI-04 demonstrated a higher detection rate for GGNs than ^18^F-FDG PET/CT. **Table 3** shows the detailed parameters in the lesions between ^18^F-FAPI-04 and ^18^F-FDG PET/CT. Upon analysis of the different ^18^F-FAPI-04 uptake parameters in all lesions, we found that the SUV_max_, SUV_mean_, SUV_index_, and TBR of ^18^F-FAPI-04 PET/CT in the lesions were higher than those of ^18^F-FDG (all *P* < 0.05).

**Table 2:** Comparison of detection rate in the lesions between Al^18^F-NOTA-FAPI-04 and ^18^F-FDG PET/CT.

	Al^18^F-NOTA-FAPI-04 PET/CT	^18^F-FDG PET/CT
Overall	58.3% (56/96)	14.6% (14/96)
<1.0cm	36.8% (7/19)	5.3% (1/19)
1.0-1.5cm	54.5% (12/22)	13.6% (3/22)
>1.5cm	67.2% (37/55)	18.2% (10/55)

**Table 3:** Comparison of tracer uptake in the lesions between ^18^F-FAPI-04 and ^18^F-FDG PET/CT.

Lesions and parameters	FAPI-04 PET/CT	FDG PET/CT	*P* value
Positive GGNs			
No. of all lesions	96	96	
Median SUV_max_	2.1 (0.4, 5.8)	1.1 (0.3, 4.6)	<0.001
Median SUV_mean_	1.4 (0.3, 3.6)	0.7 (0.2, 3.3)	<0.001
Median SUV_index_	5.6 (0.8, 29.0)	3.0 (0.6, 15.3)	<0.001
Median TBR	1.4 (0.4, 4.7)	0.6 (0.1, 3.5)	0.001
No. of GGNs <1.0cm	19	19	
Median SUV_max_	1.2 (0.4, 4.6)	0.8 (0.3, 2.8)	0.02
Median SUV_mean_	0.9 (0.3, 2.7)	0.5 (0.2, 1.3)	0.01
Median SUV_index_	2.7 (0.8, 9.7)	3.0 (0.6, 7.7)	0.42
Median TBR	0.8 (0.4, 3.9)	0.6 (0.2, 2.1)	0.06
No. of GGNs 1.0-1.5cm	22	22	
Median SUV_max_	2.0 (0.6, 4.0)	1.0 (0.5, 2.1)	0.02
Median SUV_mean_	1.4 (0.4, 2.4)	0.6 (0.3, 1.4)	0.01
Median SUV_index_	5.5 (2.0, 11.5)	2.8 (1.4, 9.3)	0.21
Median TBR	1.4 (0.5, 3.0)	0.6 (0.3, 1.9)	0.04
No. of GGNs >1.5cm	55	55	
Median SUV_max_	2.3 (0.8, 5.8)	1.2 (0.3, 4.6)	0.003
Median SUV_mean_	1.4 (0.3, 3.6)	0.7 (0.2, 3.3)	0.04
Median SUV_index_	5.9 (2.2, 29.0)	3.3 (0.6, 15.3)	0.005
Median TBR	1.6 (0.7, 4.7)	0.7 (0.1, 3.5)	<0.001

SUV_max,_ maximum standardized uptake value; SUV_mean,_ mean standardized uptake value; SUV_index,_ the ratio of SUV_max_ of lesion to SUV_max_ of contralateral normal lung paranchyma; TBR, target-to-background ratio.

### Assessment of the invasion of GGNs with ^18^F-FAPI-04 PET/CT and ^18^F-FDG PET/CT

**Figure 2:**
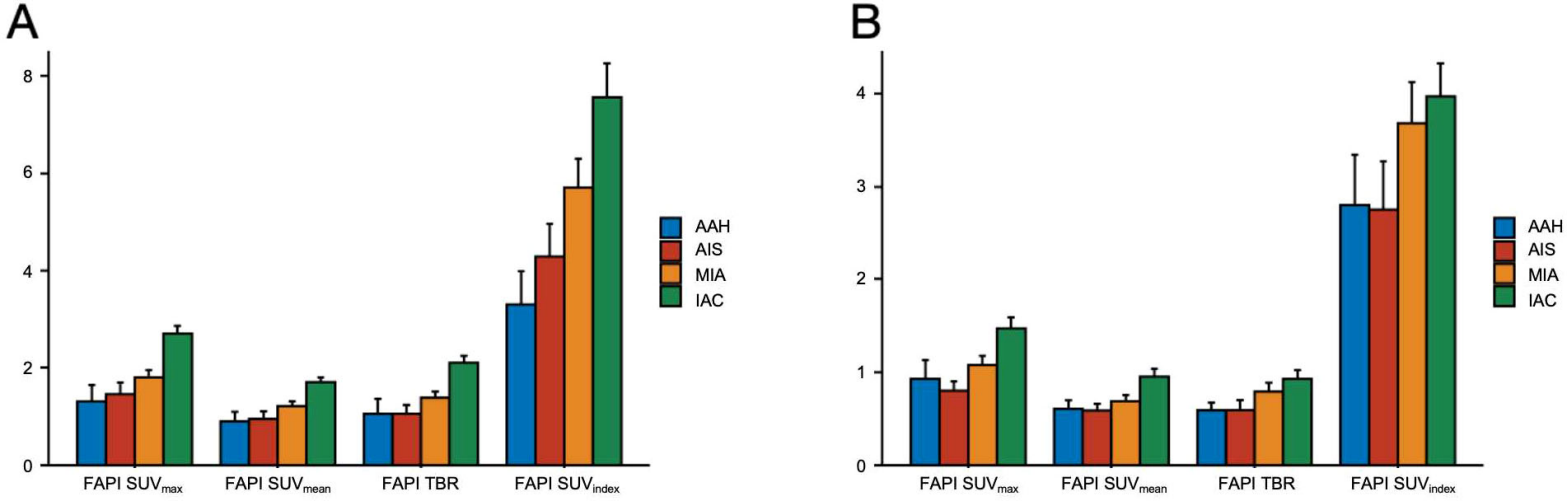
Comparison of ^18^F-FAPI-04 and ^18^F-FDG PET/CT for different pathological subtypes of GGNs. **(A)** Bar chart shows a comparison of ^18^F-FAPI-04 PET/CT for different pathological subtypes of GGNs. **(B)** The bar graph compares different pathological subtypes of GGNs evaluated by ^18^F-FDG PET/CT. FAPI, fibroblast-activation protein inhibitor; FDG, fluorodeoxyglucose; SUVmax, maximum standardized uptake value; SUVmean, mean standardized uptake value; SUVindex, the ratio of SUVmax of lesion to SUVmax of contralateral normal lung paranchyma; TBR, target-to-background ratio; AAH, atypical adenomatous hyperplasia; AIS, adenocarcinoma in situ; MIA, microinvasive adenocarcinoma; IAC, invasive adenocarcinoma.

**Figure 3:**
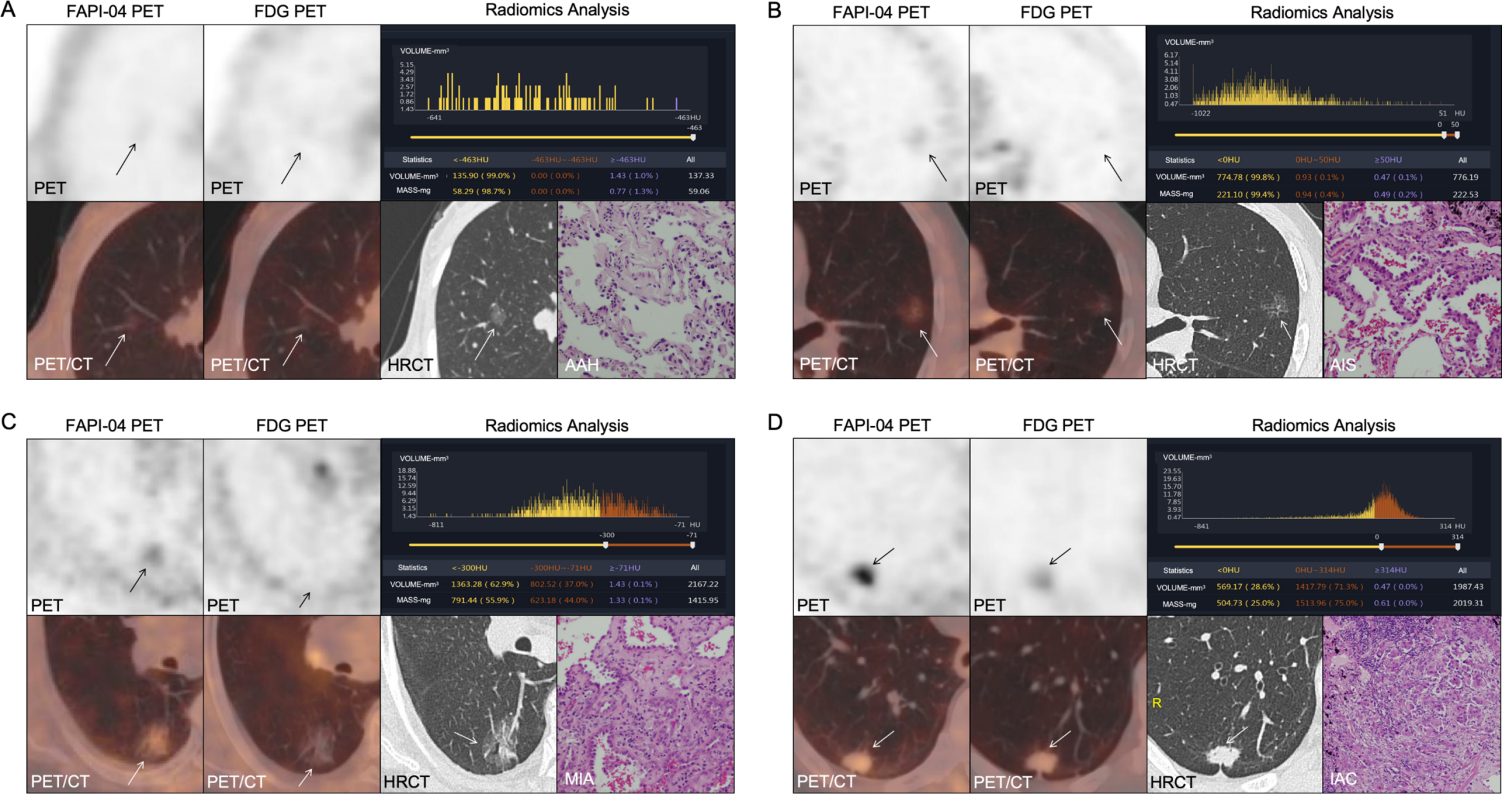
**(A–D)**^18^F-FAPI-04 and ^18^F-FDG PET/CT images, histogram distribution of CT attenuation values and pathological images of 4 different types GGNs (arrows): **(A)** AAH. **(B)** AIS. **(C)** MIA. **(D)** IAC. FAPI, fibroblast-activation protein inhibitor; FDG, fluorodeoxyglucose; AAH, atypical adenomatous hyperplasia; AIS, adenocarcinoma in situ; MIA, microinvasive adenocarcinoma; IAC, invasive adenocarcinoma.

Based on histopathologic examination and biopsy results, 96 lesions (10 AAH lesions, 14 AIS lesions, 25 MIA lesions, and 47 IAC lesions) were enrolled. **Figure 2A** illustrates a representative ^18^F-FAPI-04 PET/CT scan of four different pathological subtypes of GGNs and an ^18^F-FDG PET/CT scan of equivalent lesions (**Figure 2B**). Detailed uptake parameters for each tracer are presented in **Table 4**. ^18^F-FAPI-04 PET/CT demonstrated a higher uptake value of SUV_max_, SUV_mean_, TBR, and SUV_index_ of ^18^F-FAPI-04 PET/CT compared to ^18^F-FDG PET/CT in diagnosing GGNs (all *P* < 0.05). **Figures 3A–D** shows representative examples of four different histopathologic types.

**Table 4:** Values of parameters for different histological subtypes of GGNs.

Median of variables	Histological subtypes				*P* value
	AAH	AIS	MIA	IAC	
FAPI-04 PET/CT					
SUV_max_	1.0 (0.6, 4.6)	1.4 (0.4, 4.0)	1.9 (0.7, 3.5)	2.6 (1.2, 5.8)	<0.001
SUV_mean_	0.7 (0.4, 2.7)	0.9 (0.3, 2.4)	1.2 (0.4, 2.3)	1.7 (0.3, 3.6)	<0.001
SUV_index_	2.4 (0.8, 8.1)	4.5 (2.0, 11.4)	5.1 (1.4, 13.0)	6.6 (2.0, 29.0)	<0.001
TBR	0.7 (0.5, 3.9)	0.8 (0.4, 3.0)	1.3 (0.5, 2.9)	1.7 (0.6, 4.7)	<0.001
FDG PET/CT					
SUV_max_	0.7 (0.4,2.8)	0.7 (0.4, 1.9)	0.9 (0.3, 2.1)	1.3 (0.3, 4.6)	<0.001
SUV_mean_	0.5 (0.3, 1.3)	0.5 (0.3, 1.4)	0.6 (0.2, 1.4)	0.8 (0.2, 3.3)	0.13
SUV_index_	2.0 (1.2, 7.4)	2.3 (1.2, 9.3)	2.7 (0.6, 8.0)	3.7 (0.6, 15.3)	0.12
TBR	0.5 (0.3, 1.1)	0.5 (0.3, 1.9)	0.6(0.2, 2.1)	0.7 (0.1, 3.5)	0.18
Diameter (mm)	7.7 (5.3, 15.6)	11.5 (4.6, 15.7)	15.6 (4.9, 38.7)	21.0 (6.3, 38.3)	<0.001
CT_GGN_ (HU)	-586.9 (-730.0, -52.048)	-573.0 (-738.0, -21.3)	-485.35 (-690.0, -121.44)	-325.0 (-718.7, -10.0)	<0.001
CT_LP_ (HU)	-807.2 (-866.7, -706.8)	-834.6 (-894.0, -718.0)	-815.1 (-899.4, -719.0)	-823.6 (-894.0, -689.0)	0.58
CT_GGN-LP_ (HU)	194.7 (12.9, 800.3)	320.7 (-20.0, 761.9)	327.3 (29.0, 665.5)	478.5 (69.0, 859.2)	<0.001
pGGN	11	9	17	0	
mGGN	0	4	9	46	
Lobulaton Sign	0	5	16	39	
Spiculation Sign	0	1	10	34	
Vacuole Sign	1	2	2	12	
Plural Indentation	0	1	10	37	

AAH, atypical adenomatous hyperplasia; AIS, adenocarcinoma in situ; MIA, microinvasive adenocarcinoma; IAC, invasive adenocarcinoma; SUVmax, maximum standardized uptake value; SUVmean, mean standardized uptake value; SUVindex, the ratio of SUVmax of lesion to SUVmax of contralateral normal lung paranchyma; TBR, target-to-background ratio; CTGGN, the attenuation values of the GGN component; CTLP, the attenuation values of the normal lung parenchyma adjacent to GGN; CTGGN-LP, the difference between CTGGN and CTLP; HU, Hounsfield Unit; pGGN, pure ground-glass nodule; mGGN, mixed ground-glass nodule.

**Figure 4:**
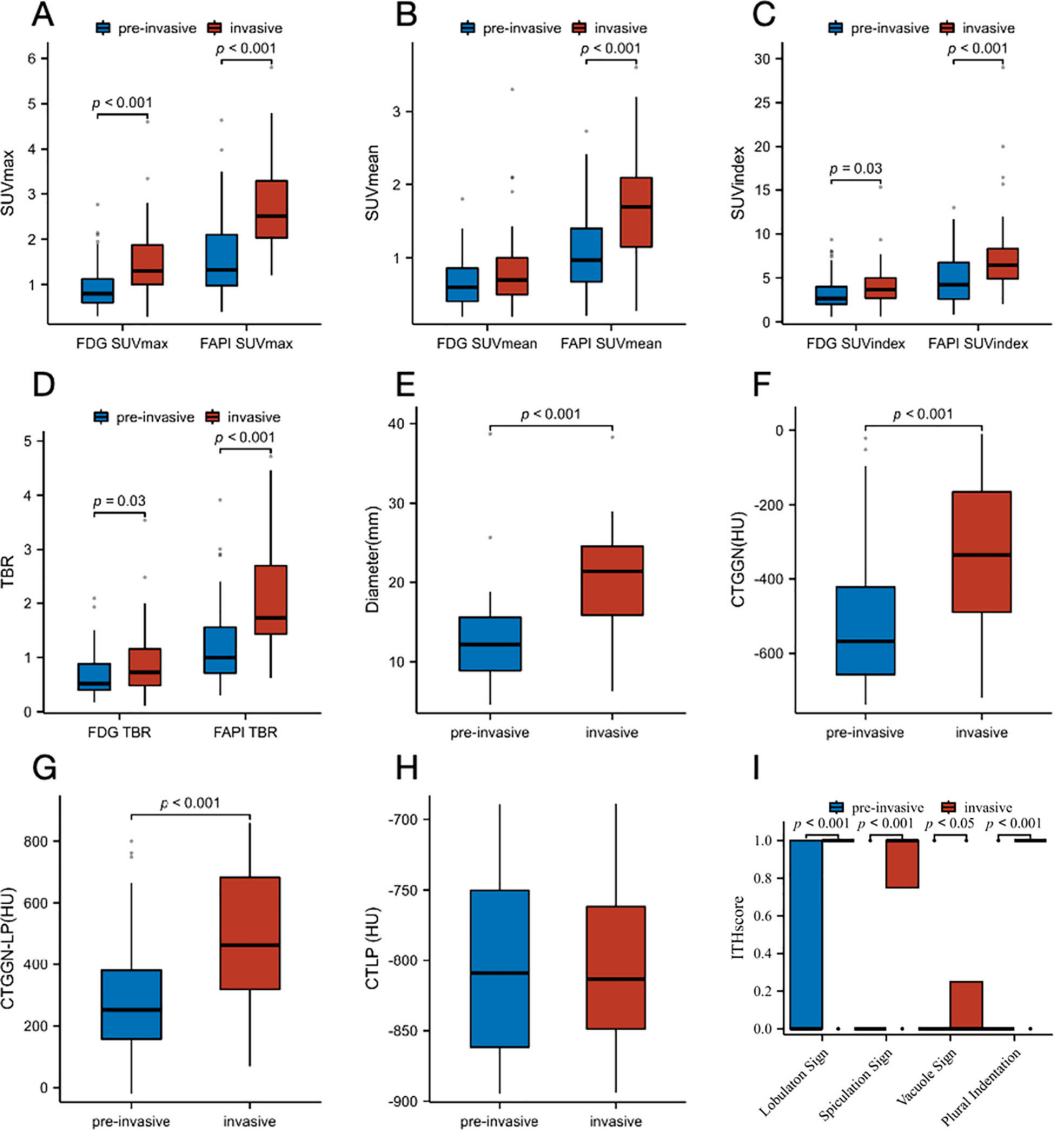
Box plots for the comparison of distribution of pre-invasive and invasive subtypes of GGNs. **(A)** SUV_max_ on ^18^F-FAPI-04 and ^18^F-FDG PET/CT. **(B)** SUV_mean_ on Al^18^F-NOTA-FAPI-04 and ^18^F-FDG PET/CT. **(C)** SUV_index_ on Al^18^F-NOTA-FAPI-04 and ^18^F-FDG PET/CT. **(D)** TBR on Al^18^F-NOTA-FAPI-04 and ^18^F-FDG PET/CT. **(E)** Diameter (mm). **(F)** CT_GGN_ (HU). **(G)** CT_LP_ (HU). **(H)** CT_GGN-LP_ (HU). **(I)** Imaging manifestations (lobulaton sign, spiculation sign, vacuole sign and plural indentation). FAPI, fibroblast-activation protein inhibitor; FDG, fluorodeoxyglucose; SUVmax, maximum standardized uptake value; SUVmean, mean standardized uptake value; SUVindex, the ratio of SUVmax of lesion to SUVmax of contralateral normal lung paranchyma; TBR, target-to-background ratio; CTGGN, the attenuation values of the GGN component; CTLP, the attenuation values of normal lung parenchyma adjacent to GGN; CTGGN-LP, the difference between CTGGN and CTLP; HU, Hounsfield Unit; ITH, intratumor heterogeneity.

The parameters of ^18^F-FAPI-04 and ^18^F-FDG PET/CT showed different diagnostic performances between the pre-invasive and invasive GGNs. Univariate and multivariate analyses were performed to analyze the association between the invasion of GGNs and clinical factors, including ^18^F-FAPI-04, ^18^F-FDG PET/CT, and CT-associated parameters. **Table 5** demonstrates plural sign (OR = 7.552, *P* = 0.015) was the independent significant factor. **Figures 4A–I** shows the comparison of pre-invasive and invasive subtypes of GGNs with all parameters. The invasive pathological subtypes showed higher uptakes than the pre-invasive subtypes. Compared with ^18^F-FDG PET/CT, ^18^F-FAPI-04 PET/CT also showed significantly higher uptake in all subtypes of GGNs (all *P* < 0.05).

**Table 5:** Univariate and multivariate analysis for the prediction of GGNs.

Variable	OR	Univariate analysis Hazard ratio (95% CI)	*P* value	OR	Multivariate analysis Hazard ratio (95% CI)	*P* value
FAPI-04 PET/CT						
SUV_max_	3.057	1.811-5.161	<0.001	1.885	0.109-7.601	0.909
SUV_mean_	5.140	2.309-11.442	<0.001	1.884	0.224-15.857	0.560
SUV_index_	1.263	1.087-1.468	0.002	0.949	0.719-1.253	0.715
TBR	3.233	1.751-5.970	<0.001	0.981	0.185-5.212	0.982
FDG PET/CT						
SUV_max_	3.523	1.613-7.692	0.002	2.942	0.116-74.881	0.513
SUV_mean_	2.611	0.969-7.040	0.05	0.574	0.013-25.625	0.242
SUV_index_	1.133	0.942-1.364	0.19			
TBR	2.106	0.942-4.773	0.08	0.111	0.007-1.683	0.113
Diameter(mm)	1.239	1.130-1.358	<0.001	1.095	0.956-1.255	0.191
CT_GGN_(HU)	1.004	1.002-1.007	<0.001	0.999	0.986-1.012	0.851
CT_GGN-LP_(HU)	1.004	1.002-1.006	<0.001	1.001	0.989-1.013	0.856
Lobulaton Sign	7.694	2.884-20.524	<0.001	0.678	0.110-4.187	0.675
Spiculation Sign	10.045	3.930-25.678	<0.001	2.083	0.495-8.769	0.317
Vacuole Sign	3.176	1.022-9.876	0.05	1.547	0.228-10.470	0.655
Pleural Indentation	14.576	5.421-39.193	<0.001	7.552	1.478-38.582	0.015

SUVmax, maximum standardized uptake value; SUVmean, mean standardized uptake value; SUVindex, the ratio of SUVmax of lesion to SUVmax of contralateral normal lung paranchyma; TBR, target-to-background ratio; CTGGN, the attenuation values of the GGN component; CTLP, the attenuation values of the normal lung parenchyma adjacent to GGN; CTGGN-LP, the difference between CTGGN and CTLP; HU, Hounsfield Unit; OR, odds ratio.

### Impacts of ^18^F-FDG and ^18^F-FAPI-04 PET/CT on the identification of the invasion of GGNs

**Figure 5:**
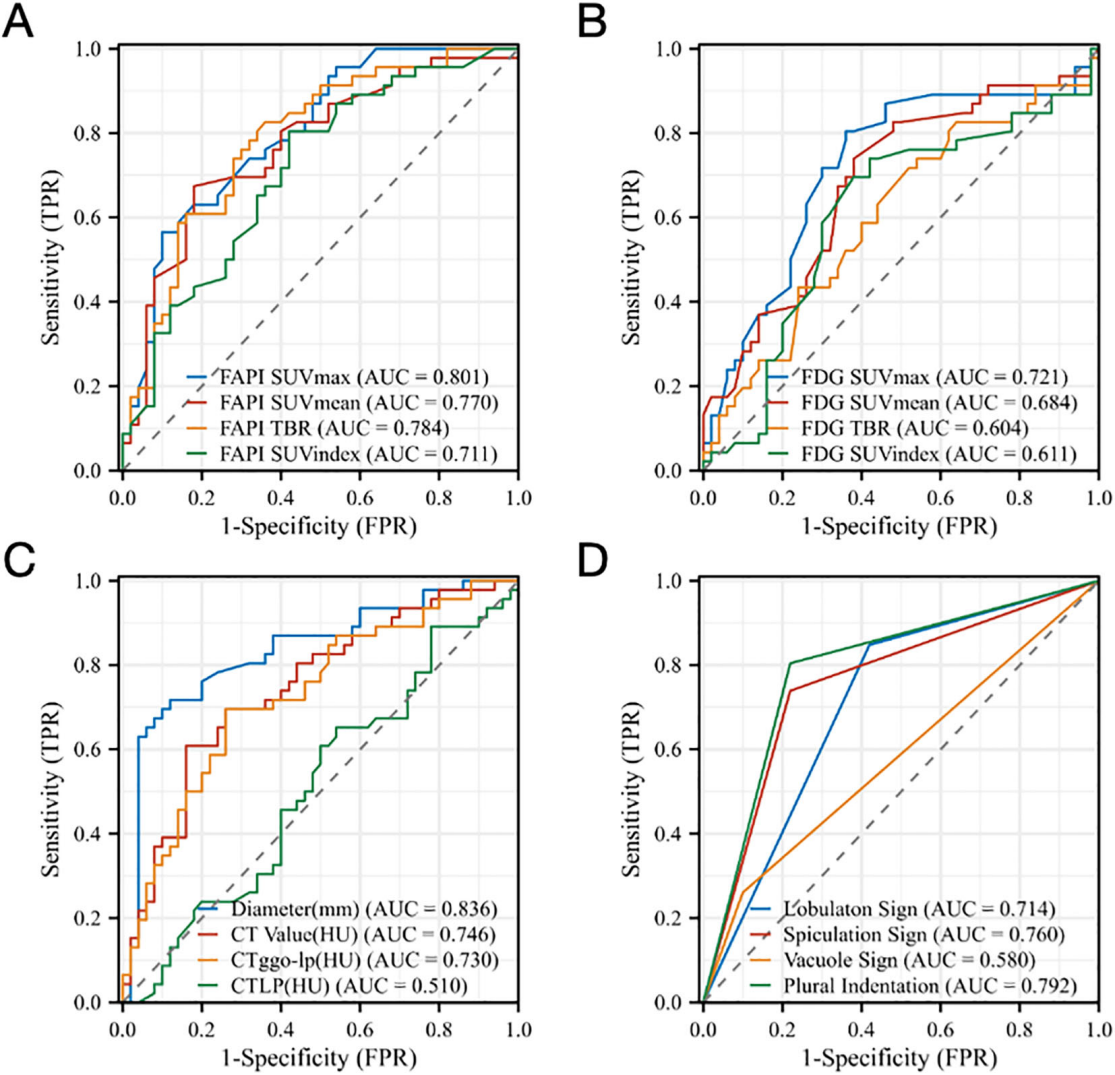
ROC analysis for different parameters **(A)** SUV_max_, SUV_mean_, SUV_index_, and TBR of Al^18^F-NOTA-FAPI-04 PET/CT, **(B)** SUV_max_, SUV_mean_, SUV_index_, and TBR of ^18^F-FDG PET/CT, **(C)** Diameter (mm), CT_GGN_ (HU), CT_LP_ (HU), CT_GGN-LP_ (HU) and **(D)** Imaging manifestations (lobulaton sign, spiculation sign, vacuole sign and plural indentation) derived from the two tracers for identifying the Invasion of GGNs. FAPI, fibroblast-activation protein inhibitor; FDG, fluorodeoxyglucose; SUVmax, maximum standardized uptake value; SUVmean, mean standardized uptake value; SUVindex, the ratio of SUVmax of lesion to SUVmax of contralateral normal lung paranchyma; TBR, target-to-background ratio; CTGGN, the attenuation values of the GGN component; CTLP, the attenuation values of normal lung parenchyma adjacent to GGN; CTGGN-LP, the difference between CTGGN and CTLP; AUC, area under the curve; TPR, true positive rate; FPR, false positive rate.

All four ^18^F-FAPI-04 PET/CT parameters showed reasonable diagnostic accuracy according to the ROC curves. Based on SUV_max_, SUV_mean_, SUV_index_, and TBR, optimal cut-off values were determined for all lesions. For ^18^F-FAPI-04 PET/CT, it is shown that the SUV_max_ AUC value for pre-invasive and invasive GGNs was 0.801, 0.770 of the SUV_mean_, 0.711 of the SUV_index_, and 0.784 of the TBR. According to ROC curve comparisons, SUV_max_ has a cut-off value of 2.5, SUV_mean_ of 1.4, SUV_index_ of 4.7, and TBR of 1.4 (**Figure 5A**).

For ^18^F-FDG PET/CT, the SUV_max_ cut-off value for noninvasive and invasive GGNs was 0.9, the SUV_mean_ was 0.6, the SUV_index_ was 2.7, and the TBR was 0.5. According to ROC curve comparisons, SUV_max_ has an AUC value of 0.721, SUV_mean_ of 0.684, SUV_index_ of 0.611, and TBR of 0.604, which suggested that the ROC curves showed ^18^F-FAPI-04 is better at identifying the invasion of GGNs than ^18^F-FDG PET/CT (**Figure 5B**).

Our data suggested that the diameters of lesions, CT_GGN_ and CT_GGN-LP_, may also relate to the invasion of GGNs. It is shown that the diameter cut-off value for pre-invasive and invasive GGNs was 16.5mm and the AUC value of 0.836, the CT_GGN_ cut-off value was -363.1HU and the AUC value of 0.746, and the CT_GGN-LP_ cut-off value was 395.0HU and the AUC value of 0.730, which showed good diagnostic accuracy. The CT_LP_ showed no diagnostic accuracy according to the ROC curve (**Figure 5C**). In addition, **Figure 5D** demonstrated that the imaging manifestations (including lobulation sign, spiculation sign, vacuole sign and plural indentation) show a certain correlation of invasion of GGNs. The AUC value of lobulation sign was 0.714, 0.760 of spiculation sign, 0.580 of vacuole sign and 0.792 of pleural indentation by ROC analysis, respectively.

## Discussion

In this study, we evaluated patients with various pathologically confirmed GGNs using ^18^F-FAPI-04 PET/CT and ^18^F-FDG PET/CT for a prospective clinical trial. This pilot study demonstrated the superior diagnostic performance of ^18^F-FAPI-04 PET/CT in GGNs with different pathological stages compared with that of ^18^F-FDG PET/CT. The underlying advantages of radiolabeled FAPI application on lung cancer have been discussed in several articles, focusing on the efficiency of detecting primary and metastatic lesions (18–20), which showed that lung cancer is a FAPI–avid tumor, and FAPI PET/CT showed some superiorities to FDG PET/CT in the detection of suspected metastases to the lymph nodes, brain, bone, and pleura but showed similar performance in the delineation of primary tumors and detection of suspected metastases in the lungs, liver, and adrenal glands.

Although the sensitivity of detecting advanced lung cancers with FAPI has been well reported, there is little information on diagnosing FAPI in GGNs. In the current analysis, we observed that ^18^F-FAPI-04 PET/CT resulted in higher SUV_max_, SUV_mean_, SUV_index_, and TBR values and better detection rates based on TBR compared to ^18^F-FDG PET/CT (24–26). Meanwhile, we found that when based on SUV_index_, ^18^F-FAPI-04 PET/CT and ^18^F-FDG PET/CT would have a reasonable detection rate. However, from a visual evaluation, it is hard to distinguish between normal tissue uptake and mild uptake of lesions in clinical practice. Our data demonstrated that ^18^F-FAPI-04 PET/CT showed some superiorities to ^18^F-FDG PET/CT in detecting primary tumors in early adenocarcinoma.

**Figure 6:**
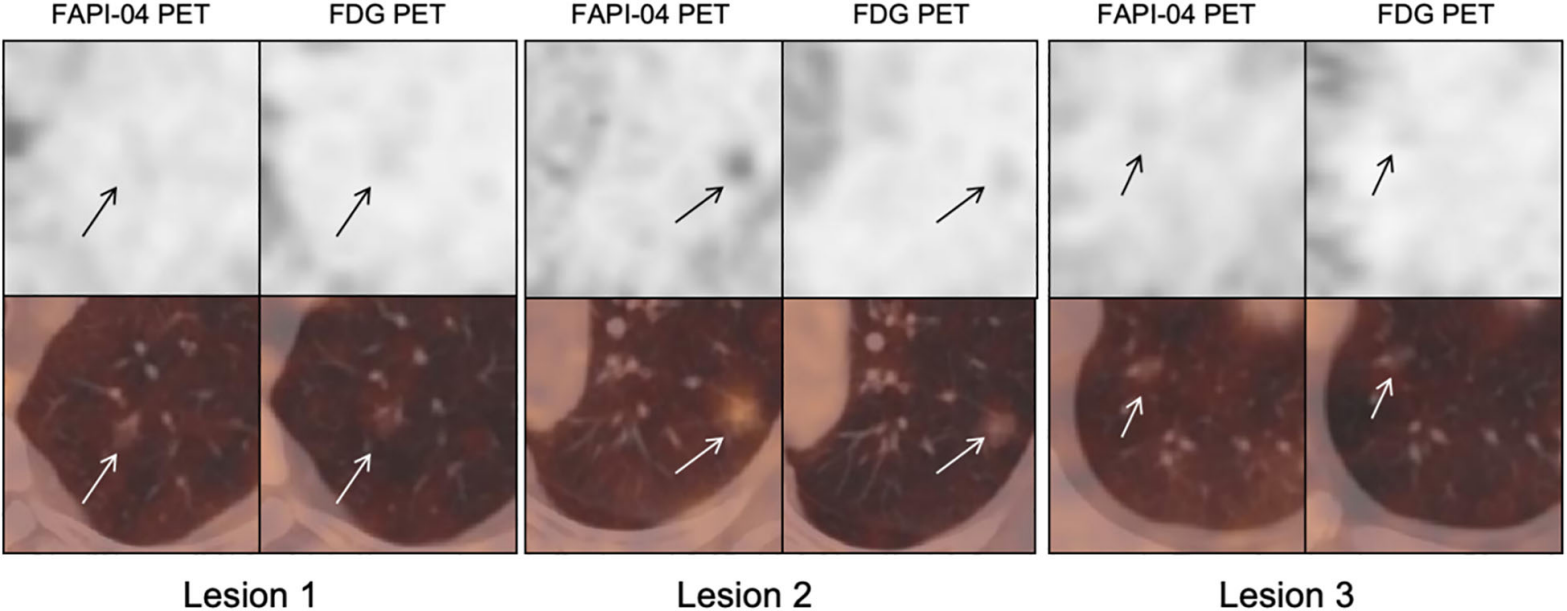
Male, 62 years old, CT showed multiple ground-glass nodules. ^18^F-FDG PET imaging showed all nodules negative uptake, while Al^18^F-NOTA-FAPI-04 PET showed an intense uptake nodule in superior lobe of left lung (arrows). FAPI, fibroblast-activation protein inhibitor; FDG, fluorodeoxyglucose.

A case report indicated that FAPI showed higher uptake in a GGN than FDG PET/CT (14). At this point, our conclusions were consistent with the previous case. In addition, our study has demonstrated that ^18^F-FAPI-04 PET/CT showed significantly different uptake values in different subtypes of GGNs and significantly higher uptake in all subtypes of GGNs compared with ^18^F-FDG PET/CT. Specific to each GGN type investigated, our study suggested that on ^18^F-FAPI-04 and ^18^F-FDG PET/CT, the invasive pathological subtypes showed higher uptakes than noninvasive ones. The latest lung cancer treatment guidelines recommend observation for patients with multiple GGNs to further develop a treatment plan (27). FAPI may help evaluate nodules that require priority removal or focus follow-up. **Figure 6** presents imaging of a specific patient with multiple GGNs, highlighting the detection of the largest nodules in the left superior lobe, which exhibited the highest SUV_max_ uptake in FAPI and was subsequently recommended for removal by the thoracic surgeon. Considering our patient-based findings, we infer that ^18^F-FAPI-04 PET/CT might guide treatments for early-stage adenocarcinoma patients with multiple GGNs. However, it is noteworthy that S. McDermott et al.’s study suggested benign GGOs, and the benign GGN subgroup demonstrated significantly higher FDG uptake at PET than malignant GGOs/GGNs (28). Awareness of this finding may prevent misinterpretation of highly ^18^FDG-avid pure GGOs/GGNs as definitively malignant, which could lead to unnecessary thoracic surgery and its associated risks.

The current study shows that ^18^F-FAPI-04 PET/CT is better at identifying the invasion of GGNs than ^18^F-FDG PET/CT. Whereas ^18^F-FDG PET/CT had an AUC value for SUV_max_ of 0.721 and SUV_mean_ of 0.684 according to the ROC curve, ^18^F-FAPI-04 PET/CT has a better value of 0.801 and 0.770. Our findings on all four parameters of the ROC curves agree with those reported by Shao et al. (29), who found that GGNs in the invasive adenocarcinoma (IAC) group showed higher SUV_max_ and SUV_index_ on FDG PET than those in the pre-invasive MIA group. Given that the growth and development of GGNs usually follow the natural progression from pre-invasive lesions (AAH, AIS, and MIA) to IAC (30), and that GGNs at different stages have different SUV uptake values (9, 31), our findings support that ^18^F-FAPI-04 PET/CT may have the potential to differentiate pathological subtypes and predict invasiveness of GGNs. The radiologic–pathologic correlation of ground-glass nodules is summarized in **Table 6**, illustrating how CT and PET characteristics reflect the histopathological spectrum from pre-invasive to invasive adenocarcinoma.

**Table 6:** Radiologic–pathologic correlation of ground-glass nodules (GGNs).

Radiologic appearance	Pathological correlation	Description/Key features	Clinical implication
Pure GGN (non-solid nodule)	Atypical adenomatous hyperplasia (AAH) / Adenocarcinoma in situ (AIS)	Homogeneous ground-glass opacity without any solid component on thin-section CT. Corresponds to pre-invasive lesions with lepidic growth pattern and absence of stromal, vascular, or pleural invasion. Typically shows low or no ^18^F-FDG and ^18^F-FAPI uptake.	Indolent behavior; suitable for active surveillance or limited resection.
Part-solid GGN (mixed GGN)	Minimally invasive adenocarcinoma (MIA) / Early invasive adenocarcinoma (IAC)	Lesion containing both ground-glass and solid components within the same nodule. The solid portion ≤5 mm indicates minimal invasion. Mild-to-moderate uptake on PET.	Represents transition from pre-invasive to invasive disease; usually requires surgical resection.
Predominantly solid nodule	Invasive adenocarcinoma (IAC)	Predominantly solid lesion with or without a residual ground-glass portion. Pathologically characterized by stromal and vascular invasion beyond lepidic growth. Shows higher ^18^F-FDG and ^18^F-FAPI uptake.	Aggressive biological behavior; lobectomy or radical resection recommended.

Additionally, Zheng et al. (9) reported diameter [odds ratio (OR), 1.159; *P* < 0.001], lobulation (OR = 2.953; *P* = 0.002), and vascular changes (OR = 3.431; *P*< 0.001) were retained as independent predictors of the IAC group. Niu et al. (8) also reported that the proportions of mixed GGN type, polygonal or irregular shape, lobulated or spiculated edge, and dilated, distorted, or cut-off bronchial sign were higher for IAC GGNs than for preinvasive GGNs, and the attenuation value of the ground-glass opacity component on CT (CT_GGO_), which showed a similar result in our study. Our study shows that the diameter cut-off value for noninvasive and invasive GGNs was 16.5mm, and the AUC value of 0.836. Our results imply that ^18^F-FAPI-04 PET/CT has the potential to identify IAC in early lung adenocarcinoma preoperatively, but it must be confirmed in further research.

The study had several limitations. First, the number of patients was small; more GGNs were required for further study. In addition, as this was a single-center study, the findings should be interpreted with caution and require validation through larger multicenter clinical trials to confirm the generalizability and robustness of the results. Second, intratumor heterogeneity and sampling bias were inherent in the histopathology and immunochemistry analysis. Another limitation is the potential influence of the partial volume effect (PVE), particularly in ground-glass nodules smaller than 1 cm in diameter. PVE may have led to underestimation of radiotracer uptake in some pre-invasive lesions. This effect is inherent to PET imaging and applies to both ^18^F-FAPI-04 and ^18^F-FDG; however, it is likely more pronounced in FDG due to its lower lesion-to-background contrast. Importantly, since both tracers were equally subject to this limitation, the observed superiority of ^18^F-FAPI-04 over FDG in differentiating invasive from pre-invasive lesions remains valid.

Despite these limitations, our results provide new evidence of the value of ^18^F-FAPI-04 PET/CT in detecting GGNs and overcoming the bias of PET/CT diagnosis in early-stage adenocarcinoma. From a practical perspective, ^18^F-FAPI-04 is currently available mainly for research use. Several academic and clinical centers in Europe, Asia, and the United States have established routine production of ^18^F-FAPI-04 under good manufacturing practice (GMP) conditions. The synthesis and quality-control procedures are similar to those of ^18^F-FDG, and the overall production cost is comparable. However, the global availability of FAPI tracers remains limited because of ongoing regulatory approvals and the absence of commercial distribution in many regions. With the expansion of multicenter clinical trials and increasing industrial collaboration, the accessibility and clinical application of ^18^F-FAPI-04 are expected to improve substantially in the near future.

Our study indicates that ^18^F-FAPI-04 PET/CT is useful for detecting early-stage adenocarcinoma. Our results show that it also outperforms ^18^F-FDG PET/CT in the detection of GGNs and can potentially be an alternative to ^18^F-FDG for detecting the invasiveness of GGNs.

## Data Availability

The datasets presented in this article are not readily available. Data, analytic methods, and study materials will not be made available to other researchers. Requests to access the datasets should be directed to PF, fupeng0451@163.com.
